# Magnitude and associated factors of postpartum depression among women in Nekemte town, East Wollega zone, west Ethiopia, 2019: A community-based study

**DOI:** 10.1371/journal.pone.0224792

**Published:** 2019-11-13

**Authors:** Muktar Abadiga

**Affiliations:** School of Nursing and midwifery, Institute of Health Sciences, Wollega University, Nekemte, Ethiopia; university of campus biomedico, ITALY

## Abstract

**Background:**

Postpartum depression is a non-psychotic disorder that happens during the first 1year after childbirth. It affects both the mother’s health and child’s development and is given significant public health concern in developed countries. However, in developing countries including Ethiopia, postnatal care is mainly concerned with obstetric problems and the baby’s health, while the psychological well-being of the mother is given little attention. Therefore, this study was aimed to assess the magnitude and associated factors of postpartum depression among women in Nekemte town, East Wollega zone, West Ethiopia, 2019.

**Methods:**

Community-based cross-sectional study was conducted on 295 postnatal women, from May 15 to June 5, 2019, in Nekemte town. The study participants were selected by a simple random sampling method and interviewed using structured questionnaires. Multivariable logistic regression was used to find the independent variables which are associated with postnatal depression. All associations between dependent and independent variables and statistical significance were measured using odds ratio at 95% confidence interval and p-value less than 0.05.

**Results:**

From the total of 295 women sampled, 287 were participated in the study. Out of these 287 women participated, 20.9% had developed postnatal depression. Unplanned pregnancy (AOR = 7.84, 95% CI: 3.19, 19.26), Being first time mother (AOR = 4.99, 95% CI: 1.54, 16.09), History of previous depression (AOR = 3.06, 95% CI: 1.06, 8.82), Domestic violence (AOR = 5.92, 95% CI: 2.44, 14.40), History of substance use (AOR = 3.95, 95% CI: 1.52, 10.30) and poor social support (AOR = 6.59, 95% CI: 2.25, 19.29) were significantly associated with postnatal depression.

**Conclusion:**

In this study, the magnitude of postnatal depression was found moderate compared to other studies. Perinatal depression screening and intervention need to be integrated with maternal health care services, especially for mothers at risk of postnatal depression.

## Background

The postpartum period is an increased time of risk for serious mood disorders [1]. Postpartum depression is a non-psychotic condition that happens during the first 1year after childbirth [2]. It usually begins within 1 month of childbirth and may continue for several months [3]. However, it may continue for 4 years after birth [4]. Postpartum depression is manifested by symptoms such as the feeling of unworthy to live, having negative thoughts about the baby, low self-worth and interest, sadness, guilt, anxiety, deeming oneself insubstantial in taking care of the baby, sleeping and eating disorders [5]. In most women, symptoms are transient; however, 10–15% of women experience a persistent form of mood disturbance [6]. Globally, the prevalence of postpartum depression ranges from 0.5% to 60.8% [7]. In developed countries, the prevalence of PPD is about 6–13% [8]. A meta-analysis, including 59 studies from North America, Europe, Australia, and Japan showed the prevalence of postpartum depression as 13% [9]. In low- and middle-income countries, the prevalence of postpartum depression is approximately 20% [10]. In Ethiopia, the prevalence of postnatal depression is about 22.4% [11].

Postnatal depression (PND) is a public health problem affecting both the mother’s health and the child’s development. It has the potential to negatively affect a new mother’s health and her ability to nurture the infant [12]. Postpartum depression can affect the mother’s capacity to care for and bond with her new-born and may react negatively towards the child. [13]. Postpartum depression can lead to poor infant feeding practices, leading to malnutrition and reduced infant growth [14, 15]. PPD can also decrease interaction and bonding between the mother and child, leading to the inadequate social, emotional and cognitive development of the child [16]. PPD occurs at a period when infant development is happening; causing children to have behavioral, cognitive, and emotional problems [17]. Postpartum depression contributes to more than 12.3% disability-adjusted life years [18].

Studies showed that the ill health of a baby, mothers of preterm baby and very low birth weight infants are at increased risk of postnatal depression [19]. Illiteracy, poor socioeconomic status, lack of social support, obstetric complication, previous history of depression, poor marital relationship, history of domestic abuse and unintended pregnancies are also contributing factors of postpartum depression [20–23]. In developed countries, PPD is given significant public health concern. However; in developing countries, it is often neglected and under-diagnosed. In Ethiopia, postnatal care is mainly concerned with obstetric problems and the baby’s health, while the social and psychological well-being of the mother is given little attention. Consequently, little is known about the magnitude of the postpartum depression and contributing factors. Therefore, this study was aimed to assess the magnitude and associated factors of postpartum depression in Nekemte town, west Ethiopia.

## Methods

### Study design and setting

This study was conducted in Nekemte town from May 15 to June 5, 2019. Nekemte town is the largest town in East Wollega Zone located at a distance of 328 kilometers from capital city Addis Ababa. This town has an estimated total population of 84,506 of whom 42,121 were males and 42, 385 were females. The total number of households in this town is about 16,901 and the number of postpartum women with an infant less than 12 months are estimated to be 2414. The community based cross-sectional study design was employed in this study. All postpartum women in Nekemte town who gave birth within 12 months before data collection were the source population and sampled postpartum women were the study population. All women who gave birth within the last 12 months prior to data collection were included in the study. Postpartum women who are eligible but not willing to take part in the study were excluded from the study.

### Sample size determination and sampling techniques

The sample size of the study was calculated using the formula for estimation of a single population proportion with the assumptions of 95% Confidence Level (CL) and marginal error (d) of 0.05. Twenty-two-point four percent (22.4%) of proportion of postpartum women with depression was taken from the previous study done in Mizan Aman town, Bench Maji zone, Southwest Ethiopia [11]. After adding a non-response rate of 10%, a total of 295 postpartum women were enrolled in the study. A simple random sampling method was used to select the study participants. First, postpartum women with an infant less than 12 months of age living in Nekemte town were identified with the help of local extension workers. The data collectors physically visited all households of the town and searched the postpartum women according to our inclusion criteria. The local extension worker assisted the data collectors in the door to door searching of postpartum women. Then, by using simple random sampling methods, a total of 295 postpartum women living in Nekemte town were recruited to be participated in the study.

### Measurement and data collection procedure

Data was collected using a validated, pre-tested structured questionnaire and a face-to-face interview was used for data collection. Edinburgh postnatal depression scale (EPDS), 3-item Kansas Marital Satisfaction Scale and 3-item Oslo Social Support Scale were used to assess postpartum depression, level of marital satisfaction and level of social support respectively. EPDS has 10 items and each item has 4 Likert scales and it has a maximum score of 30 and minimum scores of zero. Postpartum women were categorized in to depressed (total sum score ≥10) and not depressed (total sum score <10) [24]. Women with Kansas Marital Satisfaction scale score ≥17 was considered as satisfied and those <17 indicates dissatisfaction with marital status [25]. Women with 3- Items Oslo Social Support Scale score of 3–8 is considered as having poor support, 9–11 is moderate support and 12–15 is strong support [26]. Mothers were considered as a victim of domestic violence when they experienced any of physical, psychological or sexual harm within their intimate relationship. Women were considered as using substances when using any kind of addictive substance during pregnancy or after childbirth measured by yes or no items with at least one yes response. Data was collected by six trained nurses and three senior midwives as a supervisor for a duration of approximately 20 days.

### Data processing and analysis

The data were coded, checked, cleaned and entered into Epi data version 3.1 and then exported to SPSS window version 20.0 for analysis. Descriptive statistics such as frequencies and percentages were performed. Bivariate analysis was done to find an association between each independent variable with postpartum depression. Finally, multivariable logistic regression was used to find out the independent variables which influence postpartum depression. All associations between dependent and independent variables and statistical significance were measured using odds ratio at 95% confidence interval and p-value less than 0.05.

### Data quality control

The questionnaire was prepared in English language and then translated to local language Afan Oromo and then translated back to English by expertise to check for consistency. Five percent (5%) of the questionnaire was pre-tested on postpartum women living in Gimbi town which is found at about 87 kilometers distance from Nekemte town. Data collectors and supervisors were trained for two days on the clarity of tools and overall data collection procedures. Each day after completion of data collection, data collectors meeting was held and filled questionnaires were cross-checked for possible errors.

### Ethical consideration

The study was reviewed and approved by the Institutional Review Boards of Wollega University Ethical review board. A formal letter was submitted to Nekemte town administrative office and Nekemte town health bureau. After getting permission from the Nekemte town administrative office, all participants of the study were provided written consent, clearly stating the objectives of the study and their right to refuse. Participants were interviewed after clearly informing the consent and signing the consent form written in local language. After the mothers signed the written consent form, the data collectors commenced the interview. Filled out questionnaires of the study were also carefully handled and all access to results was kept strictly within the author to prevent unnecessary exposure by third parties.

## Results

### Socio-demographic characteristics

Out of the total of 295 postnatal women sampled, 287 were participated in the study; making a response rate of 97.28%. From the total of 287 postnatal women who participated in the study, 150 (52.3%) lie in the age group between 25–34 years, and the mean age of the women was 29.6 years with +/- 9.45 standard deviation. Regarding marital status of the women, the majority of the women were married and have husband, which accounts 247 (86.1%). Concerning educational status of the women, 103 (35.9%) were completed grade 9–12, and 95 (33.1%) were completed grade 1–8. The majority of the study participants were Oromo 209 (72.8%), followed by Amhara 50 (17.4%). Regarding the occupation status of the women, 112 (39.0%) were private employees followed by a daily worker, 90 (31.4%). Regarding the monthly income of women, 83 (28.9%) gets a monthly income of 500–100 EB followed by monthly income 1001–1500 EB which accounts for 76 (26.5%) (Table 1).

**Table 1:** Distribution of study participants by socio-demographic characteristics among postnatal women in Nekemte town, West Ethiopia, 2019 (n = 287).

Variables	Category	Frequency	Percentage
	15–24	71	24.7
	25–34	150	52.3
	>34	66	23.0
	Total	287	100
Marital status	Married	247	86.1
	Single	13	4.5
	Divorced	16	5.6
	Widowed	11	3.8
	Total	287	100
Educational status	Can’t read and write	35	12.2
	1–8 grade	95	33.1
	9–12 grade	103	35.9
	Diploma	38	13.2
	Degree and above	16	5.6
	Total	287	100
Ethnicity	Oromo	209	72.8
	Amhara	50	17.4
	Tigre	18	6.3
	Others	10	3.5
	Total	287	100
Occupational status	Government employee	71	24.7
	Private employee	112	39.0
	Daily worker	90	31.4
	Others	14	4.9
	Total	287	100
Monthly income	< 500 EB	44	15.3
	500–1000 EB	83	28.9
	1001–1500 EB	76	26.5
	1501–2000 EB	60	20.9
	>2000 EB	24	8.4
	Total	287	100

### Obstetrics and behavioral characteristics

From a total of 287 mothers participated in the study, 81 (28.2%) had an unplanned pregnancy, 18 (6.3%) had a history of preterm birth or death of the infant, 27 (9.4%) had experienced an obstetric complication, and 90 (31.4%) had a history of abortion. Among the respondents, 35 (12.2%) had history of previous depression, 84 (29.3%) were not satisfied with their current marital status, 68 (23.7%) had domestic violence, 38 (13.2%) had experienced stressful life event during pregnancy, and 46 (16.0%) had medical illness during pregnancy. Seventy-one (24.7%) of the study participants had poor social support, 46 (16.0%) had history of substance use, 67 (23.3%) loss job due to pregnancy, 21 (7.3%) had delivered by caesarian section and 45 (15.7%) delivered their baby at home (Table 2).

**Table 2:** Distribution of study participants by obstetrics and behavioral variables among postnatal women in Nekemte town, West Ethiopia, 2019 (n = 287).

Variables	Category	Frequency	Percentage
Pregnancy type	Planned	206	71.8
	Unplanned	81	28.2
	Total	287	100
Preterm/death of infant	Yes	18	6.3
	No	269	93.7
	Total	287	100
Obstetric complication	Yes	27	9.4
	No	260	90.6
	Total	287	100
Postnatal week	<3 months	62	21.6
	3–5 months	90	31.4
	6–8 months	85	29.6
	9–12 months	50	17.4
	Total	287	100
Number of parities	1	64	22.3
	2–4	154	53.7
	>4	69	24.0
	Total	287	100
Mode of delivery	Vaginal	205	71.4
	Caesarian section	21	7.3
	Instrumental delivery	61	21.3
	Total	287	100
History of abortion	Yes	90	31.4
	No	197	68.6
	Total	287	100
Place of delivery	Health facility	242	84.3
	Home	45	15.7
	Total	287	100
Presence of chronic illness	Yes	36	12.5
	No	251	87.5
	Total	287	100
History of previous depression	Yes	35	12.2
	No	252	87.8
	Total	287	100
Current marital satisfaction	Yes	203	70.7
	No	84	29.3
	Total	287	100
Domestic violence	Yes	68	23.7
	No	219	76.3
	Total	287	100
Jobless due to pregnancy	Yes	67	23.3
	No	220	76.7
	Total	287	100
Substance use	Yes	46	16.0
	No	241	84.0
	Total	287	100
Social support	Poor	71	24.7
	Moderate	144	50.2
	Strong	72	25.1
	Total	287	100
Stressful life event	Yes	38	13.2
	No	249	86.8
	Total	287	100
Presence of medical illness	Yes	46	16.0
	No	241	84.0
	Total	287	100

### Magnitude of postnatal depression

**Fig 1:**
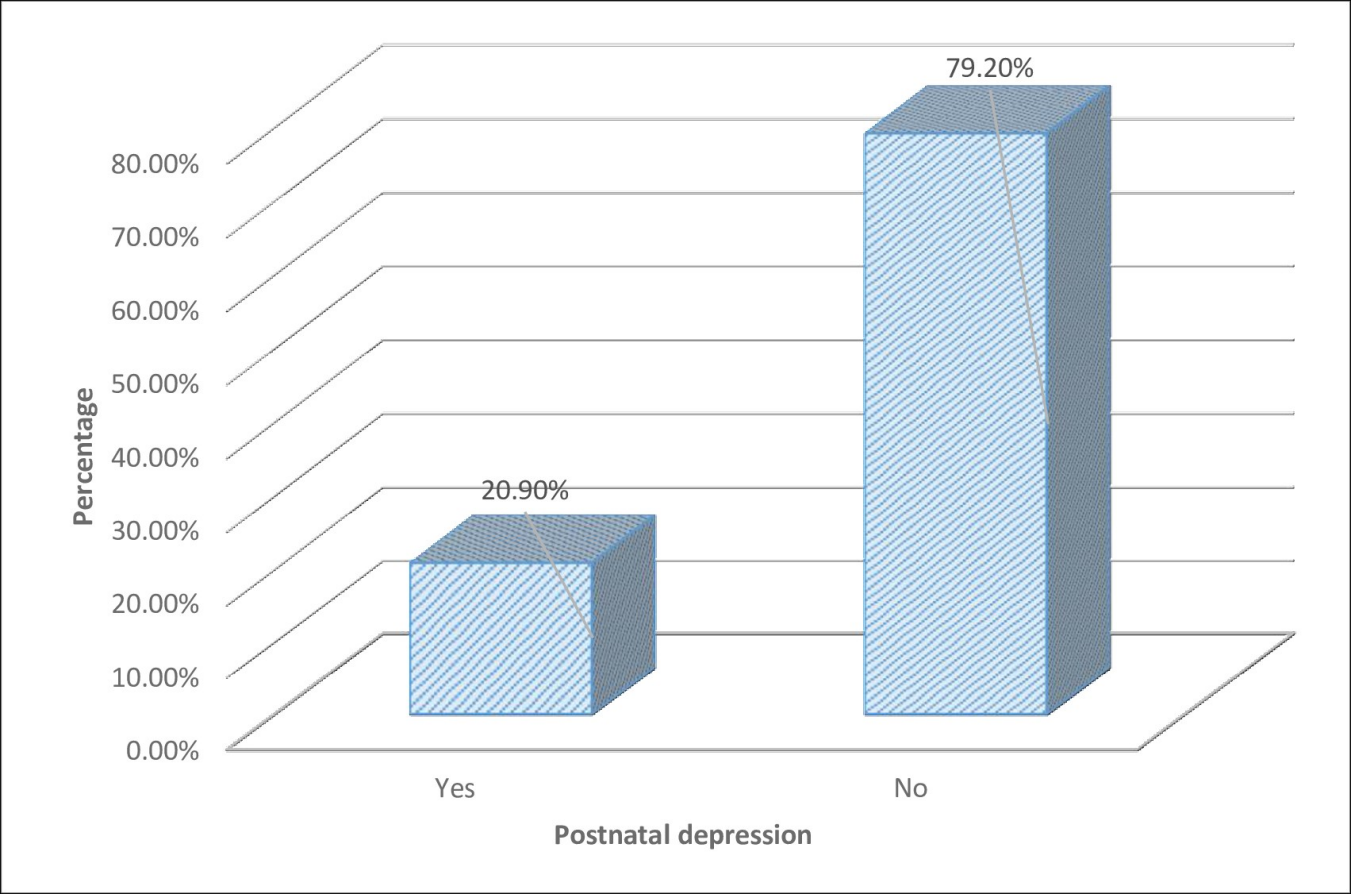
Magnitude of postnatal depression among women in Nekemte town, East Wollega zone, West Ethiopia, 2019.

The magnitude of postnatal depression was assessed by the Edinburgh postnatal depression scale (EPDS) which has 10 items. Postpartum women were categorized into depressed (total sum score ≥10) and not depressed (total sum score <10). Accordingly, out of the total of 287 study participants participated in the study, 60 (20.9%) had developed postnatal depression and 227 (79.1%) hadn’t developed postnatal depression (Fig 1).

### Bivariate logistic regression analysis

In bivariate logistic regression analysis, socio-demographic variables such as age and monthly income showed a significant association with postnatal depression. Obstetric variables such as pregnancy type, the postnatal week during an interview, history of abortion, number of parities, preterm birth or death of an infant, presence of medical illness during last pregnancy, mode, and place of delivery showed significant association with postnatal depression. Behavioral and social variables such as current marital satisfaction, domestic violence, jobless due to pregnancy, social support, history of previous depression, stressful life event and substance use showed significant association with postnatal depression (Table 3).

**Table 3:** Bivariate logistic regression analysis of factors associated with postnatal depression among women in Nekemte town, West Ethiopia, 2019 (n = 287).

Variables	Postnatal depression	COR (95%) CI	P value
	Yes (%)			No (%)
**Age of mother**				
15–24	32 (45.1%)	39 (54.9%)	5.95 (2.48, 14.26)	0.000*
25–34	20 (13.3%)	130 (86.7%)	1.12 (0.46, 2.68)	0.807
>34	8 (12.1%)	58 (87.9%)	1	
**Marital status**				
Married	51 (20.6%)	196 (79.4%)	1	
Single	2 (15.4%)	11 (84.6%)	0.69 (0.15, 3.25)	0.64
Divorced	5 (31.2%)	11 (68.8%)	1.75 (0.58, 5.25)	0.32
Widowed	2 (18.2%)	9 (81.8%)	0.85 (0.18, 4.07)	0.84
**Educational status**				
Unable to read and write	10 (28.6%)	25 (71.4%)	1.20 (0.31, 4.62)	0.79
1–8 grade	14 (14.7%)	81 (85.3%)	0.52 (0.15, 1.83)	0.30
9–12 grade	27 (26.2%)	76 (73.8%)	1.06 (0.32, 3.58)	0.91
Diploma	5 (13.2%)	33 (86.8%)	0.45 (0.10, 1.98)	0.29
Degree and above	4 (25.0%)	12 (75.0%)	1	
**Ethnicity**				
Oromo	42 (20.1%)	167 (79.9%)	1	
Amhara	11 (22.0%)	39 (78.0%)	1.12 (0.53, 2.37)	0.76
Tigre	5 (27.8%)	13 (72.2%)	1.53 (0.52, 4.53)	0.44
Others	2 (20.0%)	8 (80.0%)	0.99 (0.24, 4.85)	0.99
**Occupation**				
Government employee	12 (16.9%)	59 (83.1%)	1	
Private employee	26 (23.2%)	86 (76.8%)	1.48 (0.69, 3.12)	0.30
Daily worker	20 (22.2%)	70 (77.8%)	1.40 (0.63, 3.11)	0.40
Others	2 (14.3%)	12 (85.7%)	0.82 (0.16, 4.14)	0.81
**Monthly income**				
<500 EB	16 (36.4%)	28 (63.6%)	2.85 (0.83, 9.84)	0.09*
500–1000 EB	18 (21.7%)	65 (78.3%)	1.38 (0.42, 4.56)	0.59
1001–1500 EB	16 (21.1%)	60 (78.9%)	1.33 (0.39, 4.45)	0.64
1501–200 EB	6 (10.0%)	54 (90.0%)	0.55 (0.14, 2.17)	0.39
>2000 EB	4 (16.7%)	20 (83.3%)	1	
**Pregnancy type**				
Planned	22 (10.7%)	184 (89.3%)	1	
Unplanned	38 (46.9%)	43 (53.1%)	7.39 (3.97, 13.75)	0.000*
**Preterm/death of infant**				
Yes	5 (27.8%)	13 (72.2%)	1.49 (0.51, 4.37)	0.46
No	55 (20.4%)	214 (79.6%)	1	
**Obstetric complication**				
Yes	8 (29.6%)	19 (70.4%)	1.68 (0.69, 4.06)	0.25
No	52 (20.0%)	208 (80.0%)	1	
**Postnatal week**				
<3 months	21 (33.9%)	41 (61.1%)	2.68 (1.07, 6.75)	0.03*
3–5 months	15 (16.7%)	75 (83.3%)	1.05 (0.41, 2.68)	0.92
6–8 months	16 (18.8%)	69 (81.2%)	1.21 (0.48, 3.09)	0.67
9–12 months	8 (16.0%)	42 (84.0%)	1	
**Number of parities**				
1	23 (35.9%)	41 (64.1%)	2.95 (1.30, 6.73)	0.01*
2–4	26 (16.9%)	128 (83.1%)	1.07 (0.49, 2.31)	0.86
>4	11 (15.9%)	58 (84.1%)	1	
**Mode of delivery**				
Vaginal	42 (20.5%)	163 (79.5%)	1	
Cesarean section	7 (33.3%)	14 (66.7%)	1.94 (0.73, 5.11)	0.18*
Instrumental delivery	11 (18.0%)	50 (82.0%)	0.85 (0.40, 1.78)	0.67
**History of abortion**				
Yes	20 (22.2%)	70 (77.8%)	1.12 (0.61, 2.05)	0.71
No	40 (20.3%)	157 (79.7%)	1	
**Place of delivery**				
Health facility	45 (18.6%)	197 (81.4%)	1	
Home	15 (33.3%)	30 (66.7%)	2.18 (1.08, 4.40)	0.02*
**Presence of chronic illness**				
Yes	12 (33.3%)	24 (66.7%)	2.11 (0.98, 4.52)	0.05*
No	48 (19.1%)	203 (80.9%)	1	
**History of previous depression**				
**Yes**	13 (37.1%)	22 (62.9%)	2.57 (1.21, 5.48)	0.01*
No	47 (18.7%)	205 (81.3%)	1	
**Current marital satisfaction**				
Yes	37 (18.2%)	166 (81.8%)	1	
No	23 (27.4%)	61 (72.6%)	1.69 (0.93, 3.07)	0.08*
**Domestic violence**				
**Yes**	33 (48.5%)	35 (51.5%)	6.70 (3.59, 12.50)	0.000*
No	27 (12.3%)	192 (87.7%)	1	
**Jobless due to pregnancy**				
Yes	18 (26.9%)	49 (73.1%)	1.55 (0.82, 2.94)	0.17*
No	42 (19.1%)	178 (80.9%)	1	
**Substance use**				
Yes	23 (50.0%)	23 (50.0%)	5.51 (2.80, 10.83)	0.000*
No	37 (15.4%)	204 (84.6%)	1	
**Social support**				
Poor	34 (47.9%)	37 (52.1%)	7.35 (3.08, 17. 54)	0.000*
Moderate	18 (12.5%)	126 (87.5%)	1.14 (0.47, 2.77)	0.76
Strong	8 (11.1%)	64 (88.9%)	1	
**Stressful life event**				
Yes	11 (28.9%)	27 (71.1%)	1.66 (0.77, 3.58)	0.19*
No	49 (19.7%)	200 (80.3%)	1	
**Presence of medical illness**				
Yes	13 (28.3%)	33 (71.7%)	1.62 (0.79, 3.33)	0.18*
No	47 (19.5%)	194 (80.5%)	1	

Notes * shows significant at P-value <0.25.

### Multivariate logistic regression analysis

In the final model of logistic regression analysis, pregnancy type, number of parities, history of previous depression, domestic violence, substance use, and social support were significantly associated with postnatal depression. Mothers who had unplanned pregnancy were 7.84 times more likely to develop postnatal depression than those who had planned pregnancy (AOR = 7.84, 95% CI: 3.19, 19.26). Mothers who gave birth for the first time were 4.99 times more likely to develop postnatal depression than those who gave birth more than four times (AOR = 4.99, 95% CI: 1.54, 16.09). Mothers who had a history of previous depression were 3.06 times more likely to develop postnatal depression than those who had no history of previous depression (AOR = 3.06, 95% CI: 1.06, 8.82). Mothers who had experienced domestic violence were 5.92 times more likely to develop postnatal depression than those who had not experienced domestic violence (AOR = 5.92, 95% CI: 2.44, 14.40). Mothers who had a history of substance use were 3.95 times more likely to develop postnatal depression than those who had no history of substance use (AOR = 3.95, 95% CI: 1.52, 10.30). Mothers who had poor social support were 6.59 times more likely to develop postnatal depression than those who had strong social support (AOR = 6.59, 95% CI: 2.25, 19.29) (Table 4).

**Table 4:** Multivariate logistic regression analysis of factors associated with postnatal depression among women in Nekemte town, West Ethiopia, 2019 (n = 287).

Variables	Postnatal depression		AOR (95%) CI	P value
	Yes (%)	No (%)		
**Age of mother**				
15–24	32 (45.1%)	39 (54.9%)	0.11 (0.01, 0.99)	0.05
25–34	20 (13.3%)	130 (86.7%)	0.16 (0.07, 3.94)	0.26
>34	8 (12.1%)	58 (87.9%)	1	
**Marital status**				
Married	51 (20.6%)	196 (79.4%)	1	
Single	2 (15.4%)	11 (84.6%)	1.09 (0.13, 8.90)	0.93
Divorced	5 (31.2%)	11 (68.8%)	0.17 (0.02, 1.46)	0.10
Widowed	2 (18.2%)	9 (81.8%)	0.19 (0.01, 3.57)	0.27
**Educational status**				
Unable to read and write	10 (28.6%)	25 (71.4%)	2.16 (0.27, 16.85)	0.46
1–8 grade	14 (14.7%)	81 (85.3%)	0.50 (0.08, 3.07)	0.45
9–12 grade	27 (26.2%)	76 (73.8%)	1.67 (0.26, 10.60)	0.58
Diploma	5 (13.2%)	33 (86.8%)	0.69 (0.08, 5.56)	0.73
Degree and above	4 (25.0%)	12 (75.0%)	1	
**Ethnicity**				
Oromo	42 (20.1%)	167 (79.9%)	1	
Amhara	11 (22.0%)	39 (78.0%)	0.85 (0.24, 2.95)	0.80
Tigre	5 (27.8%)	13 (72.2%)	1.84 (0.24, 13.74)	0.55
Others	2 (20.0%)	8 (80.0%)	0.20 (0.01, 2.64)	0.22
**Occupation**				
Government employee	12 (16.9%)	59 (83.1%)	1	
Private employee	26 (23.2%)	86 (76.8%)	1.24 (0.34, 4.48)	0.73
Daily worker	20 (22.2%)	70 (77.8%)	1.30 (0.35, 4.87)	0.69
Others	2 (14.3%)	12 (85.7%)	0.82 (0.06, 10.05)	0.87
**Monthly income**				
<500 EB	16 (36.4%)	28 (63.6%)	1.19 (0.17, 8.08)	0.85
500–1000 EB	18 (21.7%)	65 (78.3%)	1.51 (0.22, 10.45)	0.67
1001–1500 EB	16 (21.1%)	60 (78.9%)	0.96 (0.15, 6.06)	0.97
1501–200 EB	6 (10.0%)	54 (90.0%)	0.61 (0.07, 4.94)	0.65
>2000 EB	4 (16.7%)	20 (83.3%)	1	
**Pregnancy type**				
Planned	22 (10.7%)	184 (89.3%)	1	
Unplanned	38 (46.9%)	43 (53.1%)	7.84 (3.19, 19.26)	0.000*
**Preterm/death of infant**				
Yes	5 (27.8%)	13 (72.2%)	0.51 (0.03, 6.88)	0.61
No	55 (20.4%)	214 (79.6%)	1	
**Obstetric complication**				
Yes	8 (29.6%)	19 (70.4%)	3.02 (0.77, 11.74)	0.11
No	52 (20.0%)	208 (80.0%)	1	
**Postnatal week**				
<3 months	21 (33.9%)	41 (61.1%)	3.02 (0.85, 10.66)	0.085
3–5 months	15 (16.7%)	75 (83.3%)	0.82 (0.23, 2.88)	0.75
6–8 months	16 (18.8%)	69 (81.2%)	1.78 (0.50, 6.22)	0.36
9–12 months	8 (16.0%)	42 (84.0%)	1	
**Number of parities**				
1	23 (35.9%)	41 (64.1%)	4.99 (1.54, 16.09)	0.007*
2–4	26 (16.9%)	128 (83.1%)	1.01 (0.38, 2.72)	0.97
>4	11 (15.9%)	58 (84.1%)	1	
**Mode of delivery**				
Vaginal	42 (20.5%)	163 (79.5%)	1	
Cesarean section	7 (33.3%)	14 (66.7%)	1.86 (0.46, 7.48)	0.37
Instrumental delivery	11 (18.0%)	50 (82.0%)	0.44 (0.13, 1.49)	0.18
**History of abortion**				
Yes	20 (22.2%)	70 (77.8%)	0.95 (0.33, 2.70)	0.92
No	40 (20.3%)	157 (79.7%)	1	
**Place of delivery**				
Health facility	45 (18.6%)	197 (81.4%)	1	
Home	15 (33.3%)	30 (66.7%)	2.09 (0.73, 5.98)	0.16
**Presence of chronic illness**				
Yes	12 (33.3%)	24 (66.7%)	2.62 (0.59, 11.54)	0.20
No	48 (19.1%)	203 (80.9%)	1	
**History of previous depression**				
**Yes**	13 (37.1%)	22 (62.9%)	3.06 (1.06, 8.82)	0.03*
No	47 (18.7%)	205 (81.3%)	1	
**Current marital satisfaction**				
Yes	37 (18.2%)	166 (81.8%)	1	
No	23 (27.4%)	61 (72.6%)	1.51 (0.52, 4.39)	0.45
**Domestic violence**				
**Yes**	33 (48.5%)	35 (51.5%)	5.92 (2.44, 14.40)	0.000*
No	27 (12.3%)	192 (87.7%)	1	
**Jobless due to pregnancy**				
Yes	18 (26.9%)	49 (73.1%)	0.97 (0.29, 3.20)	0.96
No	42 (19.1%)	178 (80.9%)	1	
**Substance use**				
Yes	23 (50.0%)	23 (50.0%)	3.95 (1.52, 10.30)	0.005*
No	37 (15.4%)	204 (84.6%)	1	
**Social support**				
Poor	34 (47.9%)	37 (52.1%)	6.59 (2.25, 19.29)	0.001*
Moderate	18 (12.5%)	126 (87.5%)	1.51 (0.52, 4.37)	0.44
Strong	8 (11.1%)	64 (88.9%)	1	
**Stressful life event**				
Yes	11 (28.9%)	27 (71.1%)	0.74 (0.17, 3.10)	0.68
No	49 (19.7%)	200 (80.3%)	1	
**Presence of medical illness**				
Yes	13 (28.3%)	33 (71.7%)	1.84 (0.57, 5.97)	0.30
No	47 (19.5%)	194 (80.5%)	1	

Notes * shows significant at P-value <0.05

## Discussion

This study examined the magnitude of postnatal depression and associated factors among mothers who gave birth within 12 months in Nekemte town, West Ethiopia. The magnitude of postnatal depression among postnatal women in this study is 20.9%. This level of postnatal depression is almost similar with study done in Udupi Taluk, India (21.5%) [27], public hospitals of Addis Ababa (23.3%) [28], Benchi Maji Zone, Ethiopia (22.4%) [29] and Kenya (18.7%) [30]. However; it is lower than a study done in Southwest Ethiopia (33.82%) [31], Vietnam (27.6%) [32], China (30%) [33] and Bangladesh (39.4%) [34]. On the other hand, the level of postnatal depression in this study is higher than study done in Netaji Subhash medical college, India (12.8%) [35], Ghana (7%) [36], Sudan (9.2%) [37] and Eastern province capital of Saudi Arabia (17.8%) [38]. The difference might be due to variation in data collection tool (different measurement scale for measuring depression), use of different cutoff points of EDPS score, sample size, sampling methods, study setting and study participant’s variation.

In this study, factors such as pregnancy type, number of parities, history of previous depression, domestic violence, substance use, and social support were significantly associated with postnatal depression. Mothers who had unplanned pregnancy were found to be more likely to develop postnatal depression than those who had planned pregnancy. This might be due to the fact that inadequate preparation for pregnancy and childbirth make the mothers unable to cope with the challenges and feel anxious. An unplanned pregnancy may also lead to economic burden and social judgment on the women which may be responsible for postnatal depression. This finding is consistent with the study done in South West Ethiopia [31], Bangladesh [34], public hospitals of Addis Ababa [28] and Benchi Maji Zone, Ethiopia [29]. In this study, mothers who gave birth for the first time were more likely to develop postnatal depression than those who gave birth more than four times This finding is consistent with a study done in Vietnam [32] and contradict with study done in Udupi Taluk, India [27]. Majorities of other studies done in different parts of countries don’t support this finding [29, 31, 34, 36, 38].

The finding of this study also showed that those who had a history of previous depression were more likely to develop postnatal depression than those who had no history of previous depression. This finding showed that postnatal depression is not only a problem which occurs during the postpartum period and indicated the importance of intervention in the prenatal period to prevent postnatal depression. This result is consistent with the study done in the Benchi Maji Zone, Ethiopia [29], China [33], Bangladesh [34], Eastern province capital of Saudi Arabia [38]. However; this result is not supported by study done in Kenya [30], Southwest Ethiopia [31], Vietnam [32] and Ghana [36]. In this study, study participants who had domestic violence were more likely to develop postnatal depression than those who had no domestic violence. The possible reason for this result is that domestic violence has an overwhelming physical, behavioral and psychological effect on the mothers and might leads to depression. Therefore, screening for domestic violence during the prenatal period could help to identify pregnant women at risk of later depression. This finding is similar to the study done in Benchi Maji zone, Ethiopia [29], Kenya [30], Bangladesh [34] and Sudan [37]. But the finding of studies done in Vietnam [32], China [33] and Saudi Arabia [38] don’t support this finding.

Mothers who had a history of substance use were more likely to develop postnatal depression than those who had no history of substance use. This might be due to the fact that substance use during the postnatal period could affect women’s emotions and behaviors, which could be responsible for the development of postpartum depression. This finding is supported by the study done in public hospitals of Addis Ababa [28] and Benchi Maji zone, Ethiopia [29]. However; this finding is not supported by study done in Kenya [30] and Bangladesh [34]. The finding of this study also illustrated that mothers who had poor social support were more likely to develop postnatal depression than those who had strong social support which is consistent with studies done in Benchi Maji zone, Ethiopia [29] and Netaji Subhash medical college, India [35]. However; this finding is not supported by many other studies [31, 32, 34, 37]. Lack of husband or family support during the stressful perinatal period would make the woman feel helpless and susceptible to postnatal depression.

## Conclusion

In this study, the magnitude of postnatal depression was found moderate compared to other studies conducted in different parts of the world. Pregnancy type, number of parities, history of previous depression, domestic violence, substance use, and social support were significantly associated with postnatal depression. Perinatal depression screening and intervention need to be integrated with maternal health care services, especially for mothers at risk of postnatal depression. Increasing access to contraceptive options, giving prompt health education to avoid substance use before pregnancy and after child birth, creating and strengthening support groups for emotional support during pregnancy and after childbirth is crucial to prevent postnatal depression.
